# Minocycline and the SPR741 Adjuvant Are an Efficacious Antibacterial Combination for *Acinetobacter baumannii* Infections

**DOI:** 10.3390/antibiotics11091251

**Published:** 2022-09-15

**Authors:** Yonas A. Alamneh, Vlado Antonic, Brittany Garry, Michael J. Pucci, Rania Abu-Taleb, Jonathan P. Shearer, Samandra T. Demons, Derese Getnet, Brett E. Swierczewski, Troy Lister, Daniel V. Zurawski

**Affiliations:** 1 Wound Infections Department, Bacterial Diseases Branch, Center for Infectious Diseases Research, Walter Reed Army Institute of Research, Silver Spring, MD 20910, USA; 2 Spero Therapeutics, Inc., Cambridge, MA 02139, USA

**Keywords:** antibiotic adjuvant, ESKAPE pathogens, antibacterial, polymyxin, therapeutic, clinical

## Abstract

Antibiotic resistance, when it comes to bacterial infections, is not a problem that is going to disappear anytime soon. With the lack of larger investment in novel antibiotic research and the ever-growing increase of resistant isolates amongst the ESKAPEE pathogens (*Enterobacter cloacae*, *Staphylococcus aureus*, *Klebsiella pneumoniae*, *Acinetobacter baumannii*, *Pseudomonas aeruginosa*, *Enterococcus* sp., and *Escherichia coli*), it is inevitable that more and more infections caused by extensively drug-resistant (XDR) and pandrug-resistant (PDR) strains will arise. One strategy to counteract the growing threat is to use antibiotic adjuvants, a drug class that on its own lacks significant antibiotic activity, but when mixed with another antibiotic, can potentiate increased killing of bacteria. Antibiotic adjuvants have various mechanisms of action, but polymyxins and polymyxin-like molecules can disrupt the Gram-negative outer membrane and allow other drugs better penetration into the bacterial periplasm and cytoplasm. Previously, we showed that SPR741 had this adjuvant effect with regard to rifampin; however, rifampin is often not used clinically because of easily acquired resistance. To find additional, appropriate clinical partners for SPR741 with respect to pulmonary and wound infections, we investigated tetracyclines and found a previously undocumented synergy with minocycline *in vitro* and *in vivo* in murine models of infection.

## 1. Introduction

ESKAPEE pathogens (*Enterococcus faecium*, *Staphylococcus aureus*, *Klebsiella pneumoniae*, *Acinetobacter baumannii*, *Pseudomonas aeruginosa*, *Enterobacter* species, and *Escherichia coli)* are a difficult set of nosocomial organisms responsible for a myriad of hospital-acquired infections. *A*. *baumannii* in particular, has been estimated to be responsible for at least a million deaths worldwide, increased stays in the hospital, and costs at over a billion dollars in patient care per year [1]. *A*. *baumannii* is a human pathogen that can often strike quickly in patients with one or multiple comorbidities and can sometimes kill susceptible patients in less than 48–72 h in cases of hospital-acquired pneumonia/ventilator-associated pneumonia (HAP/VAP) or necrotizing fasciitis [2,3,4]. In the elderly population, in critical care units, mortality rates greater than 50–60% are associated with VAP and sepsis [5,6,7,8]. Late-stage pregnant women and neonates were also found to be just as susceptible to *A*. *baumannii* with higher mortality rates when compared to other bacterial pathogens in this patient population [9,10,11]. In the U.S. military, *A*. *baumannii* also gained some notoriety from the fact that it was responsible for more than 3500 infections and was the most isolated species from U.S. troops suffering wound infections and osteomyelitis from 2004–2010 [12,13,14,15]. Moreover, there is a growing and alarming number of *A*. *baumannii* strains that are multidrug-resistant (MDR), extensively drug-resistant (XDR), and even, pandrug-resistant (PR), resistant to every FDA-approved antibiotic including colistin [16,17,18]. This growing concern motivated the World Health Organization (WHO) to cite the critical need for new antibiotics against *A*. *baumannii* in 2017 and again in 2021. Additionally, the U.S. Centers for Disease Control and Prevention (CDC) listed *A*. *baumannii* as an urgent antibiotic resistance threat in a 2019 report with an estimated 700 deaths/year and over 8500 cases per year in hospitalized patients. Overall, these combined concerns spotlight the pressing call for the development of novel therapeutics against what is becoming a formidable bacterial species for clinicians and caregivers.

Recently, a number of novel small molecules that target *A*. *baumannii* have been identified [19,20,21,22] and reviewed here [23]. Unfortunately, these potential approaches still have many hurdles to overcome and are still many years away from being an approved treatment. Alternatively, the development of adjunct therapies in combination with the current armamentarium might reach patients in need faster because adjuvants, on the whole, have less toxicity concerns than a conventional antibiotic that targets a living cell. Antibiotic adjuvants are typically small molecules with little to zero antibacterial activity on their own; however, these compounds enhance the action of antibiotics [24,25]. They do so through a variety of mechanisms, including interfering with the bacteria’s stress response, making them more vulnerable to antibiotics, or by interfering with bacterial mechanisms of antibiotic resistance. Two examples, specifically with respect to *A*. *baumannii*, include molecules such as 2-aminoimidazole (2-AI)-based compounds, which can disrupt two-component signaling and biofilms [26], and another group has shown that a subset of anthracyclines can potentiate the activity of rifampicin and linezolid [27]. Another example of antibiotic adjuvants are polymyxin or polymyxin-like molecules, which disrupt the bacterial membrane to allow better penetration of a co-administered antibiotic [28]. In doing so, a lesser amount of antibiotic is often needed, and sometimes, an antibiotic can be used that normally does not have intrinsic activity against those specific, Gram-negative bacteria. 

SPR741 is a polymyxin-B-derived molecule specifically designed to minimize, or eliminate, the dose-limiting nephrotoxicity associated with this class of antimicrobials (i.e., polymyxin B and colistin). SPR741 has a reduced positive charge (three positive charges versus the five found in polymyxin B and colistin) and lacks the highly lipophilic fatty acid side chain present in the natural polymyxins—these are the two structural features complicit in the observed clinical nephrotoxicity [29]. The reduction in charge and lipophilicity render SPR741 incapable of killing Gram-negative bacteria at clinically relevant concentrations, but the retained positive charge is sufficient to disrupt the Gram-negative outer membrane [30].

Previously, we tested a combination of SPR741 and rifampin as an effective combination against *A*. *baumannii* in an *in vivo* mouse model [31]. Others have shown the efficacy of SPR741 against other Gram-negatives with many different antibiotic combinations *in vitro* [32,33]. These previous studies and our own pre-evaluation of other classes of antibiotics helped facilitate the selection of minocycline as a clinically relevant, partner drug for further testing *in vitro* and *in vivo*. Herein, we present the results of the testing of the synergistic effects of SPR741 with minocycline against *A*. *baumannii* AB5075 *in vitro* and in a murine pulmonary and soft tissue wound model of infection. 

## 2. Results

### 2.1. In Vitro Analysis

The minimal inhibitory concentrations (MICs) for SPR741 and minocycline were determined for AB5075 using standard methods, and the individual minimal inhibitory concentrations (MICs) of SPR741 and minocycline were greater than 64 µg/mL and 0.5 µg/mL, respectively. The MIC for SPR741 was high (>64 µg/mL), but this was expected for an adjuvant molecule that does not have clinically relevant antibacterial activity on its own. The combination of the two molecules was then assessed, and our results showed a synergism between SPR741 and minocycline, resulting in an MIC of 0.125 µg/mL, a 40-fold increase in activity, as summarized in Table 1.

**Figure 1:**
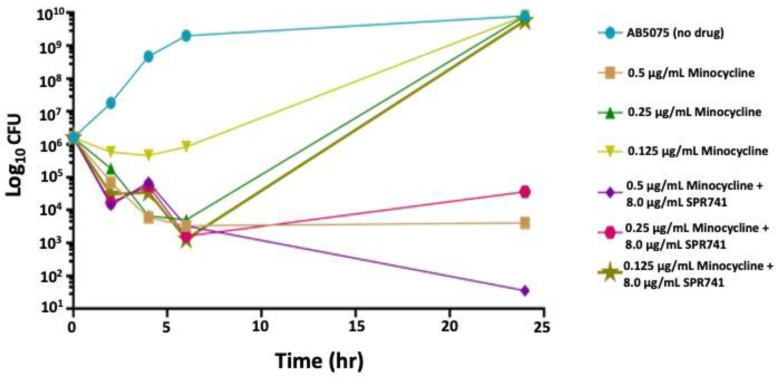
AB5075 was grown overnight in CAMHB, then subcultured in CAMHB for 2 h. Cultures were inoculated 1:10 in CAMHB alone or with 8.0 µg/mL SPR741, 0.5 µg/mL minocycline, 0.25 µg/mL minocycline, 0.125 µg/mL minocycline, or both SPR741 and minocycline at these respective concentrations. Time points were taken at 0, 2, 4, 6, and 24 h and samples plated for CFUs. The data presented are the average of three separate experiments, performed in duplicate.

To understand the effect on bacteria over time and whether this synergistic effect was bacteriostatic or bactericidal, time–kill assays were performed against AB5075 using 8.0 µg/mL SPR741 and minocycline, at three different doses: 0.5, 0.25, and 0.125 µg/mL (1×, ½×, and ¼× the MIC). Dosing with minocycline alone at 0.5 µg/mL and 0.25 µg/mL resulted in an ~3 log_10_ decrease in the number of bacteria, but that number rebounded by 24 h post-treatment (Figure 1). The lowest minocycline dose had a bacteriostatic effect for the first 6 h post-dosing, and then, the number of bacteria rose to the control levels at 24 h, like the other minocycline doses. However, with the combination of 8.0 µg/mL SPR741 and 0.5 µg/mL minocycline, we observed additive effects with the two therapeutics (3.5 × 10^1^ CFU) in comparison to 4.1 × 10^3^ CFUs for minocycline alone, and the bacteria did not display the rebound effect. Instead, the growth of AB5075 was only partially restored over the course of 24 h with a 0.25 µg/mL dose and not restored at all with the 0.5 µg/mL dose of minocycline (Figure 1). This promising synergy indicated the necessity for more than one dose over time, but also suggested that, at a higher dosage of minocycline, this drug combination has bactericidal effects, where the bacteria had difficulties recovering post-exposure. Additionally, these data suggested an *in vivo* assessment was warranted.

### 2.2. Pulmonary Infection Analysis

We previously determined that the *A. baumannii* strain AB5075 was more suitable for animal model experiments because this more virulent XDR strain could infect the host in three different animal models (*Galleria*, mice, rats), and with pulmonary infection, the bacteria disseminated into the bloodstream, resulting in mortality consistently at two days post-inoculation [34]. In a pilot study using our murine pulmonary model of *A*. *baumannii* infection, we initially evaluated three doses of minocycline (1.0, 2.5, and 5.0 mg/kg) two times per day (BID) delivered by the intraperitoneal (i.p.) route and followed animals for 3 days. Based on these experiments, the dose of minocycline of 1.0 mg/kg BID was determined to be optimal for further testing and evaluation of SPR471 co-administration because minocycline alone at this dose protected the animals 50% or less. In contrast, the larger doses resulted in >50% animal survival even without SPR741.

**Figure 2:**
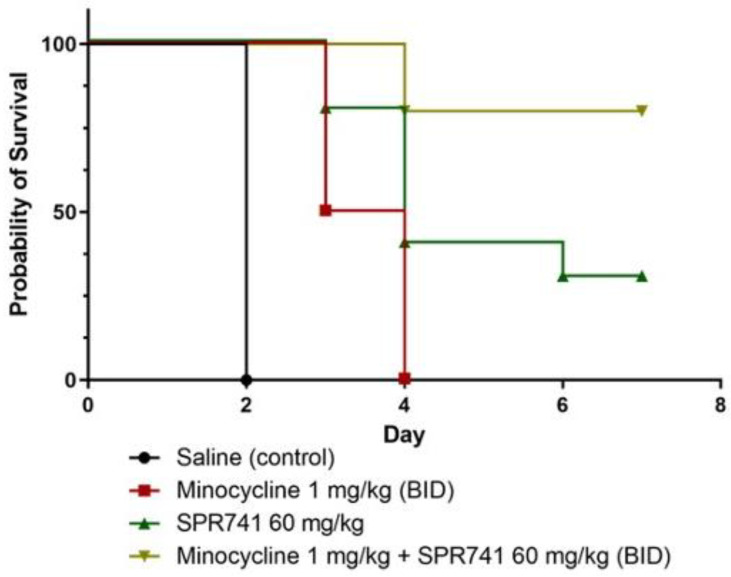
In a survival model of *A. baumannii*-infected mice, n = 10 were inoculated with 5.0 × 10^6^ CFUs of AB5075 via the intranasal route, and subsequently, animals were dosed with sterile saline (black circle), minocycline 1.0 mg/kg BID (red square), 60 mg/kg SPR741 BID (green triangle), or the combination of minocycline 1.0 mg/kg and SPR741 60 mg/kg BID (yellow triangle). This representative experiment was repeated twice (n = 20). All groups were statistically significant (*p* < 0.05) when compared to each other via the Mantel–Cox test (Graphpad Prism).

In the next series of experiments, mice treated with combination therapy, minocycline 1.0 mg/kg BID) + SPR741 60 mg/kg BID (10 mice per group), were compared to three control groups: (1) saline—an untreated group, where 100% of these mice succumbed to infection by 48 h; (2) the negative control group—animals received minocycline alone (1.0 mg/kg BID) in which all of the animals died by Day 4 post-infection; (3) the negative control group—SPR471 alone group—animals that received 60 mg/kg SPR471 BID, which resulted in 35% survival over the course of the week. In comparison to the control groups, for the group that received combination therapy minocycline (1.0 mg/kg BID) + SPR741 60 mg/kg BID, 85% of the infected mice survived for one week (Figure 2), clearly demonstrating the benefits of the combination therapy. 

**Figure 3:**
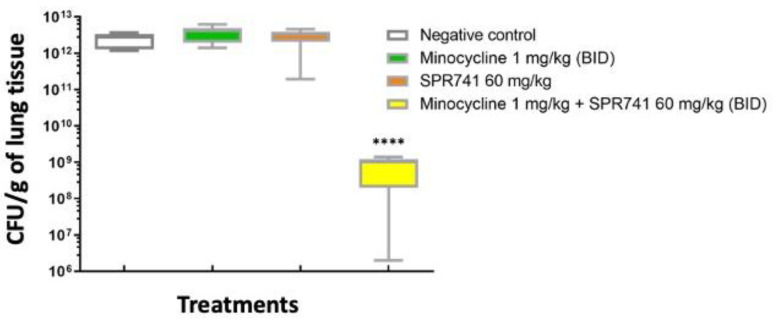
Box and whiskers plots of the log_10_ CFU/g of lung tissue on Day 2 post-inoculum. Mice were treated with minocycline 1.0 mg/kg BID (green box), SPR741 60 mg/kg BID (red box), the combination of these doses (yellow box), or sterile saline (vehicle alone, control, white box) for 2 days. Boxes show median and interquartile ranges, while whiskers represent the 95% confidence interval (CI). Groups were compared via the Mann–Whitney U-test. The combination of drugs was statistically significant when compared to the other groups (**** represent *p <* values of 0.0001). These data were pooled from two biological replicates with at least 5 mice per group, with 10 mice total per test condition.

Once survival results were obtained and repeated, we wanted to evaluate the efficacy of combination therapy (minocycline 1.0 mg/kg + SPR741 60 mg/kg) on bacterial burden. The same experimental design and groups were executed as before with the exception of a timed euthanasia on Day 2, the height of infection [34], before any of the untreated animals succumbed to infection. After euthanasia, lung tissue was removed, homogenized, and plated onto selective media for enumeration of bacterial burden. Here, the bacterial burden results supported the survival results, where the combination of SPR741 and minocycline showed the largest decrement of the bacterial numbers, the median being 3.0 log_10_ CFUs less when compared to the vehicle-alone control (*p* < 0.0001). In comparison, the median for minocycline alone, Saline control, and SPR741 alone displayed similar results without a statistically significant change (Figure 3). 

### 2.3. Wound Infection Model

**Figure 4:**
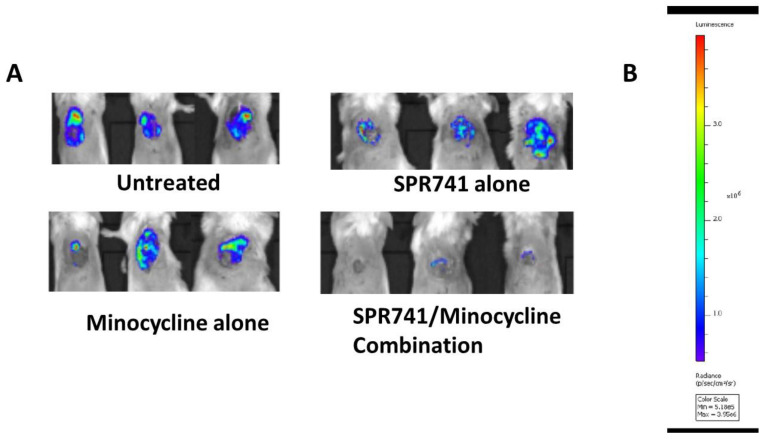
Bioluminescence of the infected wound bed. The bioluminescence of AB5075::lux was measured (in photons/s/cm2/steradian) for each mouse wound by using an IVIS. BALB/c mice (n = 10) were pretreated with cyclophosphamide and infected topically in the wound bed with 5.0 × 10^4^ CFUs of AB5075::lux. Mice were treated i.p. with minocycline, SPR741, or the combination twice a day for three days. On Day 6, animals were imaged using the IVIS to measure the radiance of each group. (**A**) Examples of each group—untreated control, SPR741 (60 mg/kg BID), minocycline (1.0 mg/kg), and combination—are shown. (**B**) Legend for radiance (quantity of bioluminescence units). Pictured are exemplar images of wound bioluminescence of mice from each group on Day 6 post-infection.

We previously used *in vivo* imaging (IVIS) to longitudinally track *A*. *baumannii* infection development in excisional soft tissue wounds using a bioluminescent version of AB5075 (AB5075::lux) [35]. The number of photons illuminated by AB5075::lux (radiance) was used for the indirect quantification of bacteria in the wound bed. Over the 6 days of follow-up, mice were treated i.p. with minocycline, SPR741, or the combination twice a day for three days with dosages as previously described in the pulmonary model of infection (Figure 2 and Figure 3). IVIS images were collected daily for the entire length of the experiments. Similar to the model of lung infection, the combination of SPR741/minocycline was superior to each therapeutic when administered alone. In the wounds of control animals, untreated controls, the number of bacteria was significantly higher than in SPR741 alone and minocycline alone (Figure 4). The radiance as measured in the group that received combination therapy minocycline (1.0 mg/kg BID) + SPR741 (60 mg/kg BID) was significantly lower in comparison to the untreated control, at all time points (Figure 4).

We confirmed the efficacy of combination therapy on bacterial burden by performing a separate set of experiments measuring the bacterial burden of animals (n = 10 mice/group) by the traditional method of counting colonies, the CFUs of the wound bed. Animals were treated at the same dose/schedule, and timed euthanasia was performed at 48 h post-infection as before. Here, the bacterial burden results supported the bioluminescence results. Untreated controls had almost 1.0 × 10^13^ CFU/g in the wound tissue. In contrast, groups treated with SPR741 60 mg/kg BID and minocycline 1.0 mg/kg BID alone had about 2–3 log_10_ less bacteria, but still had a significantly higher number of bacteria in the wound bed in comparison to the combination therapy of SPR741 60 mg/kg + minocycline 1.0 mg/kg (~5.0 × 10^8^) (*p* < 0.0001). The combination therapy in comparison to untreated animals was a dramatic reduction of approximately 5 log_10_ less bacteria, and this statistically significant difference was also captured by the IVIS and bioluminescence data (Figure 4). 

## 3. Discussion

**Figure 5:**
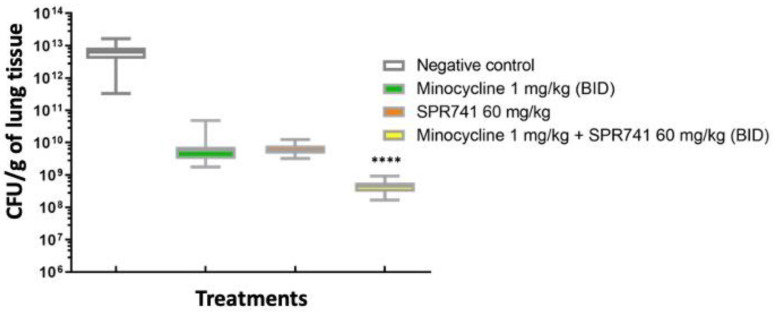
Box and whiskers plots of the log_10_ CFU/g of wound tissue on Day 2 post-inoculum. Mice were treated with minocycline 1.0 mg/kg BID (green box), SPR741 60 mg/kg BID (red box), the combination of these doses (yellow box), or sterile saline (vehicle alone, control, white box) for 2 days. Boxes show median and interquartile ranges, while whiskers represent the 95% confidence interval (CI). Each group was compared to the untreated control group via the Mann–Whitney U-test. **** represents statistical significance (*p <* values of 0.0001). These data were pooled from two biological replicates with at least 5 mice per group, with 10 mice total per test condition.

In the current study, we built upon previously reported results [31] and continued to evaluate the efficacy of the potentiator molecule SPR741 as an antibiotic adjuvant against XDR *A*. *baumannii.* Here, the combination of SPR741 and minocycline (a more clinically relevant antibiotic than the previously tested rifampin) was evaluated in a series of standard *in vitro* tests and in two *in vivo* murine models of infection, pulmonary and excisional wound. The main objective was to ascertain if the previously determined dose of SPR741 at 60 mg/kg BID could also work synergistically with minocycline and potentiate its efficacy against an XDR *A*. *baumannii* strain. We demonstrated that a combination of minocycline 1.0 mg/kg BID and SPR741 at 60 mg/kg BID significantly improved the survival of mice in a model of lethal lung infection (Figure 2). Moreover, in the wound infection model with the same doses, we observed a significant decrease in the number of bacteria in the wound bed during treatment and after the treatment stopped (Figure 4 and Figure 5). These results highlight the potential clinical utility of this combination to address both HAP/VAP and skin soft tissue infections (SSTIs).

Previously, in a proof-of-concept study, we demonstrated that SPR741 potentiates the efficacy of rifampin [31]. Due to some characteristics of rifampin such as the short half-life and the ease of spontaneous mutations that arise from treatment both *in vitro* and *in vivo* [36,37], we pivoted to minocycline as a partner as this antibiotic is still FDA-approved, but has better clinical utility. Minocycline works against both Gram-positive and Gram-negative bacteria and still has robust coverage against a number of different infection types including those seen in *A*. *baumannii*-infected patients [38,39,40,41,42]. A growing body of literature also supports the use of minocycline against *A. baumannii* infections alone or in combination with other polymyxins such as colistin and polymyxin B [41,42,43,44,45,46,47,48]. In particular, this synergy was also seen clinically [45,46]. Therefore, it is not surprising that we observed this synergistic activity with SPR741 *in vitro* and *in vivo* as well because it is also a polymyxin-like molecule. Because of minocycline’s lipophilic structural domains, it has increased tissue penetration, increased antibacterial activity, and a longer half-life, making it easier to dose in comparison to some other tetracyclines [38,39,40,41,42]. That said, ervanacycline and omadacycline, newer FDA-approved tetracyclines, also have activity against many *A*. *baumannii* isolates [49,50], and each could be a potential partner for SPR741 if the bactericidal activity and the lack of rebound growth we observed with the drug combination (Figure 1) are conserved across this whole antibiotic class. 

Like other reports, our *in vitro* data support the use of minocycline against *A. baumannii*. We observed dose-dependent bacteriostatic effects of minocycline alone. An MIC dose (0.5 μg/mL) resulted in 4.0 × 10^3^ CFUs of bacteria after 24 h of exposure. Importantly, when combined with SPR741 (8.0 μg/mL), we observed an increase in the minocycline efficacy and a 1–2 log_10_ difference between the groups. At 24 h, we observed a 2-log_10_ difference in the combination of minocycline at a ½xMIC dose and a 5-log_10_ difference at a 1xMIC dose (Figure 1). Our *in vitro* results further suggest that there is a threshold at which SPR741 can be successful in potentiating minocycline’s efficacy, and these limitations will have to be investigated for each antibiotic partner. However, it is clear that the combination can help to lower the dose of the partner tetracycline and seems to prevent the rebound of bacterial growth in this case (Figure 1). 

Two hypotheses for why the bacteria do not rebound are as follows: one, it could be that SPR741, in damaging the outer membrane, could also be disrupting the efflux pumps responsible for pumping out the minocycline, and therefore, the drug accumulates and keeps killing bacteria over time. A second idea is that the facilitated entry of minocycline by SPR741 treatment allows the molecule to bind the 30S ribosome target with a lesser concentration, and then, the additional drug could be hitting other secondary targets. Possibly, those targets are other non-coding RNA molecules involved in bacterial survival and stress responses [51,52]. Of course, both of these mechanisms of action could be occurring simultaneously and independently of one another, which explains the dramatic kill observed *in vitro* (Figure 1) and *in vivo* (Figure 3 and Figure 5). 

Our study has some limitations. For one, the present study only evaluated a combination of SPR741 and minocycline against a single strain of *A*. *baumannii*, our model strain AB5075. In our previous work, we evaluated a subset of diverse *A. baumannii* strains with SPR741, so we know it has activity across many isolates [31]; however, our colleagues have recently developed a larger diversity set, which also includes more recent isolates from around the world and more tetracycline-resistant strains [53], which could be evaluated in the future. Additionally, expanding our work to other Gram-negatives such as *Klebsiella pneumoniae* is warranted given that the synergy of minocycline and polymyxins is also observed with this species [54]. 

Another limitation is that we only evaluated the synergy of these drugs in murine models. For an SSTI clinical indication, swine is a better model, as their skin more closely resembles human skin [55], and drugs that are successful in swine models have a >89% chance of reproducing the results in humans [56]. Lastly, with respect to SSTI, in the future, we should also evaluate the rate of healing. Time to wound closure is typically a parameter we measure in our murine wound model [57], but for this study, we ran out of time and funding with respect to this project and could not fully complete all aspects of our typical evaluation of an antibacterial.

In conclusion, more preclinical investigations of SPR741 with different antibiotic combinations and different Gram-negative bacterial species are merited given this work. It is hoped other laboratories pursue this line of research. However, *in vivo* studies to date have been limited [31,58], and to our knowledge, this is the only study that has evaluated SPR741 with minocycline *in vivo*. Given the known toxicities associated with the tetracycline class, and minocycline in particular [59], the combination of minocycline with SPR741 offers an attractive approach for future clinical applications given that SPR741 was successful in a Phase 1 clinical trial [60] and because the minocycline dosage could be lowered. The data presented here are encouraging with respect to the use of SPR741 and other polymyxins in combination with minocycline and could become crucial in the fight against XDR bacteria, which has become a global problem poised to reach epidemic proportions. 

## 4. Materials and Methods

### 4.1. Bacterial Strains and Growth Media

All work was carried out under BioSafety Level II or II plus conditions. AB5075 and its antibiotic susceptibilities have been described previously [34]. Routine growth and strain maintenance was carried out in lysogeny broth (LB, Lennox formulation) with agar on plates or in liquid media. MIC tests and time–kill assays were performed using cation-adjusted Mueller–Hinton broth (CAMHB).

### 4.2. In Vitro Assays of Efficacy

The MIC was determined using an overnight culture of bacteria that was diluted to a starting inoculum of ~1.0 × 10^4^ CFU/mL in CAMHB and assessing turbidity after 24 h of incubation at 37 °C and exposure to SPR741, minocycline, or the combination of the molecules according to CLSI standards [61]. Time–kill assays were performed as previously described [31]. Briefly, AB5075 was grown overnight in CAMHB at 37 °C, then subcultured into CAMHB the next morning and grown at 37 °C until log phase growth (OD_600_ = ~0.5). Cultures were inoculated into CAMHB alone or supplemented with 8.0 µg/mL SPR741, 1.0 µg/mL minocycline, or both. A bactericidal effect was defined as a 3 log_10_ reduction or greater in CFU/mL compared with the original inoculum; a bacteriostatic effect was defined as up to a 3 log_10_ reduction in CFU/mL compared with the original inoculum.

### 4.3. Murine Pulmonary Model of Infection

Our animal use protocol, 16-BRD-48S, was approved by the Institutional Animal Care and Use Committee (IACUC) at the Walter Reed Army Institute of Research (WRAIR). The protocol was followed accordingly for all subsequent animal experiments. Six-week-old female BALB/c mice (Charles River, Wilmington, MA, USA) were housed 4–5 animals per cage and allowed access to food and water ad libitum throughout the experiment. The model has been previously published [31], but briefly, mice were rendered neutropenic, via i.p. administration of 150 mg/kg and 100 mg/kg cyclophosphamide in sterile saline on Day -4 and Day -1, prior to infection (Day 0), respectively. Then, AB5075 was grown overnight, subcultured (1:100), and grown to ~2.0 × 10^8^ CFU/mL in LB broth. Mice were anesthetized with oxygenated isofluorane immediately prior to intranasal inoculation with 25 µL of bacterial cultures, corresponding to 5.0 × 10^6^ CFU. The cell concentration of the suspension was confirmed by serial dilution and plating on LB agar.

Following infection, mice were administered with i.p. injections of sterile saline (negative control), minocycline, SPR741, or a combination of these two molecules starting at 4 h post-infection and then BID for Days 1, 2, and 3. Four hours post-inoculum was chosen as the first dose because we had previously shown that after four hours, the bacteria have proliferated almost 1.0 log_10_ CFU/g lung tissue [31,34].

In contrast to our previous study [31], we followed mouse survival for one week post-infection (out to Day 7). Animal morbidity was scored twice daily for six days using a system evaluating mobility (0 = normal movement, 1 = movement upon stimulation, 2 = absence of movement), coat condition (0 = normal coat, 1 = ruffled coat), and conjunctivitis (0 = absence of conjunctivitis, 1 = conjunctivitis), as previously described. Mice were humanely euthanized according to the protocol as they became exceedingly moribund and their clinical scores increased. 

To assess CFU burden in the lungs, mice were humanely sacrificed at 4 h or on Day 2 post-infection by an injection of ketamine (100 mg/kg) and xylazine (10 mg/kg) followed by a euthanasia procedure according to our protocol. Lungs were removed, and each sample was processed using a sterile homogenizer in sterile PBS. Samples were serially diluted 10-fold, and each dilution was plated onto LB plates in triplicate containing ampicillin to kill any resident lung flora and select for *A*. *baumannii*. Plates were incubated overnight at 37 °C and enumerated the following day.

### 4.4. Murine Wound Model of Infection

Six-week-old female BALB/c mice, weighing 14–20 g, were used for this model (Charles River, NCI, MD). The animals were maintained and used in accordance with protocol 16-BRD-48S, as above. All mice received sterile dry chow and water ad libitum, were housed in groups of five in sanitized cages on sterile paper bedding, and provided environmental enrichment. The model was conducted in this study similar to what was previously published [35,57].

In this study, four groups consisting of ten mice per group were used. To establish infectious mice were rendered neutropenic, all mice received 150 mg/kg of body weight and 100 mg/kg cyclophosphamide via i.p. injections on Day -4 and Day -1, respectively. On Day 0 (the day of infection), mice were anesthetized by intraperitoneal injection of ketamine (100 mg/kg) and xylazine (10 mg/kg) prior to performing the dermal wounding procedure, then 25 µL of bacterial cultures, corresponding to 5.0 × 10^6^ CFUs of AB5075-lux [34], were administered directly onto the center of the wound. Tegaderm™ dressing was then applied over the wound, and animals were singly caged according to the protocol. The cell concentration of the inoculum suspension was verified by serial dilution and plating on LB agar prior to infection. 

At four hours post-infection, groups were treated with sterile saline (negative control), 1.0 mg/kg minocycline BID, 60 mg/kg SPR741 BID, and the combination of minocycline 1.0 mg/kg and 60 mg/kg SPR741 BID for 3 days. Four hours was chosen here as a starting point for treatment as, similar to the lung model above, we showed that at 4 h post-infection, the bacteria grew about 0.5–1.0 log_10_ [57]. An in vivo imaging system (IVIS Lumina XR, Perkin-Elmer, Waltham, MA, USA) was used to evaluate burden via the bioluminescence of inoculated bacteria, and images were collected daily. 

We also evaluated the bacterial burden via the CFUs of the wound bed. Mice were sacrificed on Day 2 post-infection by injecting them with a dose of 100 mg/kg ketamine and 10 mg/kg xylazine. The wound beds were collected, and each sample was placed in 2 mL of sterile PBS in a 14 mL conical tube and homogenized (TissueRuptor; Qiagen Sciences, Inc., Germantown, MD, USA). Serial 10-fold dilutions of the homogenate were manually plated onto LB agar. Plates were incubated overnight at 37 °C, and then, the CFUs were enumerated the next day.

### 4.5. Statistics

All graphs and statistics were calculated using GraphPad Prism 6.0, software. For the time–kill assay, each group was compared to each other using a two-way ANOVA test. The survival curves were plotted using the log-rank (Mantel–Cox) test, and each group was statistically significant when compared to each other (*p*-values < 0.05). For CFU/g lung tissue, each group was compared via the Mann–Whitney U-test to one another. All results were considered significant if *p* < 0.05. 

## Figures and Tables

**Table 1:** MICs determined for minocycline, SPR741, and the combination of molecules.

Bacteria	Minocycline (µg/mL)	SPR741 (µg/mL)	SPR741 + Minocycline (µg/mL)	Fold-Change
AB5075	0.5	>64	0.0125	40

## Data Availability

Data will be made publicly available on the https://cdmrp.army.mil website.

## Disclaimer

Material has been reviewed by the Walter Reed Army Institute of Research. There is no objection to its presentation and/or publication. The opinions or assertions contained herein are the private views of the author, and are not to be construed as official, or as reflecting true views of the Department of the Army or the Department of Defense. Research was conducted under an IACUC-approved animal use protocol in an AAALAC International—accredited facility with a Public Health Services Animal Welfare Assurance and in compliance with the Animal Welfare Act and other federal statutes and regulations relating to laboratory animals.
